# Microbiota-Derived Extracellular Vesicles Detected in Human Blood from Healthy Donors

**DOI:** 10.3390/ijms232213787

**Published:** 2022-11-09

**Authors:** Béatrice Schaack, Thomas Hindré, Nyamekye Quansah, Dalil Hannani, Corinne Mercier, David Laurin

**Affiliations:** 1 CNRS, UMR 5525, VetAgro Sup, Grenoble INP, TIMC, Université Grenoble Alpes, 38000 Grenoble, France; 2 CEA, CNRS, IBS, Université Grenoble Alpes, 38044 Grenoble, France; 3 Etablissement Français du Sang, Département Scientifique Auvergne Rhône-Alpes,38000 Grenoble, France; 4 INSERM U1209 & CNRS UMR 5309, Institute for Advanced Biosciences, Université Grenoble Alpes, 38000 Grenoble, France

**Keywords:** extracellular vesicles (EVs), outer membrane vesicles (OMVs), membrane fusion, red blood cell concentrates, lipopolysaccharide (LPS), OmpA, gut microbiota

## Abstract

The microbiota constitutes an important part of the holobiont in which extracellular vesicles (EVs) are key players in health, especially regarding inter- and intra-kingdom communications. Analysis of EVs from the red blood cell concentrates of healthy donors revealed variable amounts of OmpA and LPS in 12 of the 14 analyzed samples, providing indirect experimental evidence of the presence of microbiota EVs in human circulating blood in the absence of barrier disruption. To investigate the role of these microbiota EVs, we tracked the fusion of fluorescent *Escherichia coli* EVs with blood mononuclear cells and showed that, in the circulating blood, these EVs interacted almost exclusively with monocytes. This study demonstrates that bacterial EVs constitute critical elements of the host–microbiota cellular communication. The analysis of bacterial EVs should thus be systematically included in any characterization of human EVs.

## 1. Introduction

Compared to their 30 trillion cells, human bodies contain an average of 39 trillion microbial cells, among which an impressive variety of Archaea, Fungi, Protozoa, and Bacteria [1]. These microbial cells affect the health, mood, and ability of the host to respond to certain medication. In particular, bacteria in the gut microbiota were recently shown to play a crucial role in the development, homeostasis, and fine-tuning of the immune system [2,3]. Immunological interactions between humans and their gut microbiota rely, for an important part, on living bacteria, and the influence of gut microbiota on various physiological and pathological settings is now clearly established [3,4]. Bacterial cells communicate with their host and other bacteria through direct contact. They use the secretion of soluble products such as metabolites (e.g., short-chain fatty acids), lipoglycans, quorum sensing peptides, nucleic acids, proteins, and bacterial extracellular vesicles [5].

All types of cells from all kingdoms of life produce extracellular vesicles (EVs) [5,6]. The diameter of these vesicles ranges from 30 to 1000 nm. Their composition depends on both the type of cell they originate from and their mechanism of biogenesis. The main known role of EVs from all kingdoms of life is to facilitate intercellular communication by transporting molecules such as lipids, nucleic acids, proteins, sugars, and metabolites [5,7].

Produced by Gram-negative bacteria, outer membrane vesicles (OMVs) constitute a particular sub-type of EVs. OMVs are natural proteo-liposomes whose double leaflet membrane, composed of phospholipids, glycolipids, lipopolysaccharides (LPS) and membrane proteins, constitutes a vesicle containing bacterial metabolites, cytosolic proteins, and nucleic acids [8]. It has been estimated that one single bacterial cell secretes ~10 vesicles during the exponential growth phase [9]. We estimated that given that the human gut contains ~4 × 10^13^ bacterial cells [1], the human microbiota may thus produce ~4 × 10^14^ bacterial EVs, which represent approximatively ~200 mg of lipids, and ~120 mg of proteins based on our characterization of *E. coli’s* OMVs (see companion paper).

Within the intestine, bacterial OMVs regulate the communication between prokaryotic species through the delivery of toxins and nucleic acids [8] and play several in vivo functions related to both intra- and inter-kingdom intercellular communications and signaling [6,9,10,11]. In particular, OMVs have been shown to help digest the host intestinal mucosa and to penetrate gut epithelial cells by using micropinocytosis, clathrin-mediated endocytosis, caveolin-mediated endocytosis, or fusion with the plasma membrane at raft-enriched spots [6,9]. They were also described as able to breach the tight junctions joining the human intestinal epithelial cells in the context of patients with intestinal barrier dysfunction [10]. In healthy humans, the disruption of epithelial cell tight junctions by OMVs [12] suggests that OMVs produced by the intestinal microbiota could (i) diffuse from the intestine into the blood circulation and directly interact with blood immune cells and (ii) be disseminated throughout the body via the bloodstream to reach distant organs whose function can be modulated by the content of OMVs. The journey of fluorescent OMVs was demonstrated in vitro and in vivo in mice in pioneering articles [13,14].

The challenging recovery of human gut microbiota OMVs coupled with gut microbiota complexity limits the detailed analysis of these fine interactions. In this report, we overcame these limitations by directly characterizing the EVs’ content of therapeutic grade blood products from several healthy donors. Using both biophysical and biochemical techniques, we demonstrated the presence of Enterobacteriaceae OMVs in a large majority of those samples. We also investigated the interactions established between such OMVs and human blood mononuclear cells by using fluorescently labeled *E. coli* OMVs that we produced (see companion paper) and demonstrated that they almost exclusively interacted with monocytes from normal human blood. We also highlighted, for the first time, the complex nature of blood EVs that include bacterial vesicles as well as human exosomes and microvesicles.

## 2. Results

### 2.1. Ex Vivo Demonstration of the Presence of Enterobacteriaceae OMV Components in Human Red Blood Cell Concentrates

**Figure 1:**
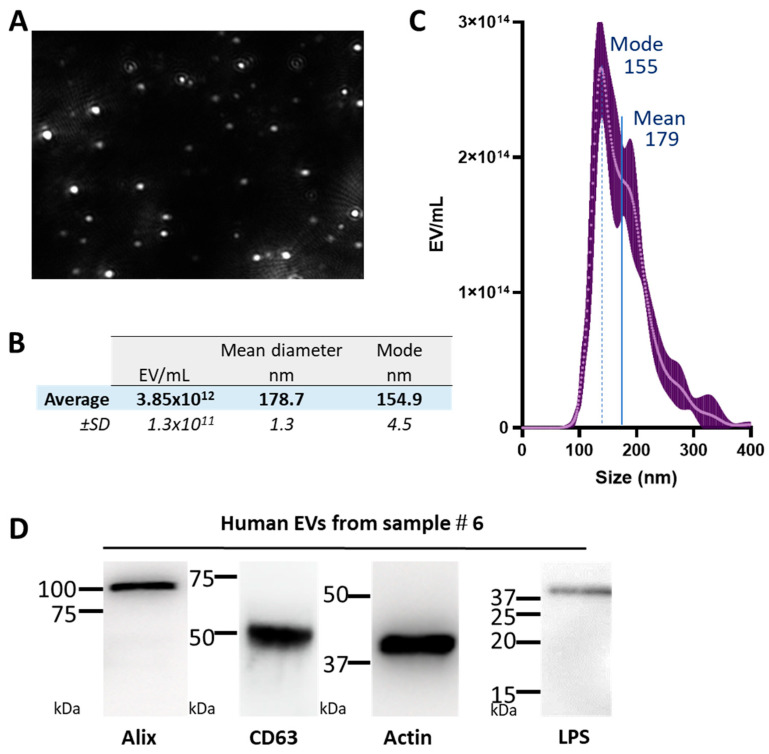
EVs from the RBC concentrates of healthy donors display heterogenous diameters and are LPS-positive. (**A**) Image extracted from a nanoparticle tracking analysis (NTA) video. EVs were counted and their diameter was measured based on their Brownian motion. (**B**) The table shows an example of the results obtained with the sample further reported as being analyzed by flow cytometry. The mean and mode of five video acquisitions performed for the NTA measurements are shown ±SD. (**C**) Histogram of the distribution of the diameter of the EVs. (**D**) Immunoblot detection of Alix, Actin, CD63, and LPS in the RBC EVs. Experiments conducted with the EVs from blood donor EV#6 (see Table 1).

Based on the observation that EVs of bacterial origin may enter the systemic circulation in patients with intestinal barrier dysfunction [10], we tested whether bacterial EVs could also be detected in the blood of healthy donors. To this aim, we isolated EVs from human red blood cell (RBC) concentrates obtained from 14 healthy donors and assessed the presence of OMVs in the resulting samples. These blood products were qualified as therapeutic products and were thus bacteria-free. Preservation of RBCs requires the presence of plasma and each analyzed blood cell concentrate (~300 mL/donor, free of platelets, and with a limited number of leukocytes), thus containing 5% to 15% of plasma. We separated the EVs from the RBC concentrates by successive centrifugations in order to eliminate the cells and then recover EVs. The resulting vesicles were filtered at 0.22 µm to eliminate any potential live cells. We then analyzed their diameter and concentration by nanoparticle tracking analysis (NTA) (an example of EVs from donor #6 is shown in Figure 1A,B; Table 1 describes this cohort more precisely). The obtained donor #6’s EVs were heterogenous in diameter, ranging from about 80 to 350 nm, with an average diameter of 178 ± 1.3 nm and a major peak at 155 nm (Figure 1B,C). Table 1 describes the characteristics of the 14 blood and EV samples, the mean diameter of the EVs was 171 nm, with a major peak (mode) at 156.1 nm. In comparison, pure *E. coli* OMVs had a smaller diameter, in the range of 30 to 52 nm (see companion paper). Immunoblot analysis of RBC EVs showed the presence of Alix, a cytosolic protein that plays a critical role in EV biogenesis; CD63, a tetraspanin highly enriched in exosomes; actin, which is an abundant cytoskeleton protein in RBCs, and the vesicles they produce (Figure 1D). Together, these analyses confirmed the presence of human EVs. More strikingly, we also detected LPS with an apparent molecular weight close to 40 kDa (Figure 1D). This size corresponds to the technical data of the manufacturer. Therefore, it corresponds to smooth LPS containing lipid A, the inner core, the outer core, and the O-antigen. The presence of LPS thus confirmed that bacterial products smaller than 200 nm in diameter were present in the EVs.

**Figure 2:**
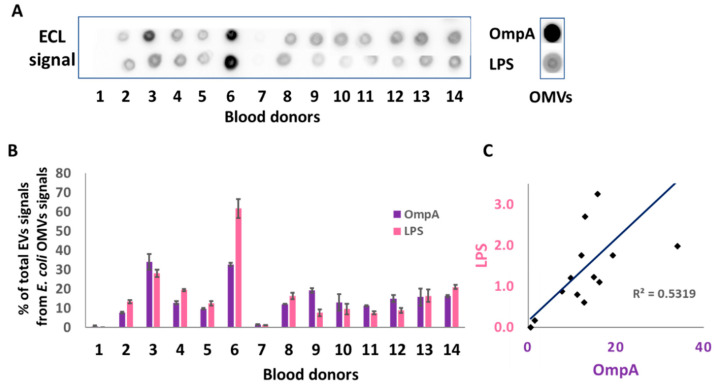
The OmpA protein and the LPS contents of RBC-EV extracts varied between healthy donors. (**A**) Dot blot of 8 µg of lipids per spot from RBC-EV extracts of 14 healthy donors and from the pure *E. coli* OMVs. The OmpA protein and the LPS contents were revealed using antibodies specific to *E. coli* OmpA or LPS followed by goat HRP-conjugated secondary antibodies, and finally by enhanced chemiluminescent detection (ECL). (**B**) Relative quantitation of the dot blot signals presented in (A) using ImageLab after establishing the OmpA and LPS signals of the *E. coli* OMVs as 100% references. The columns represent the mean values ± SD calculated from three experiments. We verified the linearity of the ECL signals for both the OmpA (R^2^ = 0.8987) and LPS (R^2^ = 0.8856) immunostainings. (**C**) Correlation between the LPS and OmpA signals for the 14 donors (R^2^ = 0.5319).

The presence of LPS is not sufficient to attest to the presence of bacterial vesicles. LPS molecules could be present in the blood as micelles [15]. We thus investigated the presence of a second typical marker of the Enterobacteriaceae membrane (i.e., OmpA), which is the major porin of the Enterobacteriaceae outer membrane. OmpA is a true transmembrane protein and has never been found as a soluble protein [16]. We dotted EV quantities corresponding to 8 µg of lipids per sample for both the extracts of blood EVs and *E. coli* OMVs on nitrocellulose membranes, which were then incubated with antibodies specific to LPS or OmpA. The analysis was performed on extracts of blood EVs from 14 donors. We used OMVs isolated from pure cultures of *E. coli* as the positive and relative control. We detected LPS signal in 12 out of 14 RBC-EV samples. The only RBC-EV negative samples were EV#1 and EV#7 (Figure 2A). These observations thus showed that bacterial EVs are frequently present in the blood of healthy donors and that they most probably originate from Gram-negative Enterobacteriaceae of the gut microbiota. Quantitation of the observed signals allowed for the calculation of the relative percentages in both OmpA and LPS in each blood EV extract compared to pure *E. coli* OMVs (Figure 2B). These results showed inter-donor variability and revealed three main profiles: (i) the blood EV extracts from donors #1 and #7 were almost devoid of OmpA and LPS; (ii) most blood samples contained low OmpA and LPS concentrations compared to pure *E. coli* OMVs, with mean relative percentages ranging from 10 to 20% of the *E. coli* OMV content (Figure 2B); (iii) EVs from both the EV#3 and EV#6 samples were particularly enriched in LPS (30 and 65% of the *E. coli* OMV content, respectively) and in OmpA (above 30% of the *E. coli* OMV content in both cases). A positive correlation (R^2^ = 0.5319) was established between the LPS and the OmpA signals (Figure 2C), confirming the detection of these two major components of the membrane of OMVs produced by Enterobacteriaceae. Together, these results strongly suggest that the quantity of OMVs in circulating human blood varies according to the composition in Gram-negative bacteria in the various microbiota, and in particular, the gut microbiota.

### 2.2. E. coli Fluorescently Labeled OMVs Are Capable of Fusion with Monocytes from Normal Human Blood

**Figure 3:**
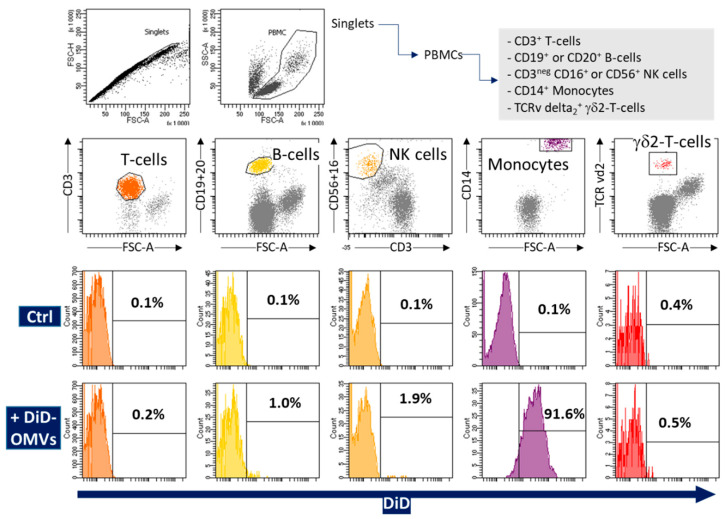
Fluorescently labeled *E. coli* OMVs fuse with human monocytes from healthy blood donors. *E. coli* OMVs were labeled with DiD lipophilic dye and incubated with PBMCs for 18 h before flow cytometry analysis. Singlet events and the morphology of PBMCs based on FSC-A/SSC-A parameters were gated. Then, lymphocyte and monocyte subtypes were identified by CD3^+^ (T-cells), CD19^+^ or CD20^+^ (B-cells), CD56^+^ or CD16^+^ population CD3^neg^ (NK-cells), and TCRv delta2^+^ (γδ T-cells variant delta2) expression. The % of DiD-positive cells is shown for the respective populations with a defined threshold relative to the background (Ctrl) measured on cells that were not incubated with DiD-labeled OMVs. These graphs are representative of two experiments providing similar data.

Observing the presence of Enterobacteriaceae OMVs in human RBC concentrates, we next sought to investigate their ability to interact with human cells. Because such OMVs cannot easily be isolated from blood samples, we used OMVs isolated from a culture of *E. coli*. These OMVs were labeled with the lipophilic marker DiD. They were incubated with peripheral blood mononuclear cells (PBMCs) purified from healthy donors at a ratio of 5 × 10^4^ OMVs per cell. After 18 h, we analyzed the cell subpopulations according to their expression of blood lineage markers by flow cytometry (Figure 3). We tested several cell populations including monocytes, T-cells, B-cells, NK-cells, and the γδ unconventional T-cells expressing the δ TCR variant 2 (a subpopulation that accounts for almost 60% of γδ T cells in peripheral blood [17]). Remarkably, under our experimental conditions, monocytes became fluorescent, with 91.6% of positive cells within this subpopulation (Figure 3). In contrast, none of the other studied PBMC sub-populations exhibited changes in fluorescence, hence demonstrating that *E. coli* OMVs fused almost exclusively with human blood monocytes but not with other PBMCs.

## 3. Discussion

The presence of bacterial proteins and bacterial membrane components has been described in all body compartments in the absence of bacteria. In addition, bacterial DNA was reported in blood [18,19], urine [20], and brain tissue [21], despite these being deemed germ-free. These bacterial elements have not been correlated to any bacterial vector. In these studies, the mediator was not specified. Metabolites, nucleic acids, proteins, and LPS micelles could either be secreted by the bacteria or released following bacterial death. Nevertheless, Turken et al. recently described extracellular vesicles bearing LPS [10] by electron microscopy in the context of a leaky gut. In addition, OMVs from *Bacteroides thetaiotaomicron* were shown to translocate through the intestinal epithelium in vitro and in vivo and reach various systemic tissues in mice [13]. Chronopoulos and Kalluri also stressed that bacterial EVs were identified as long range “hormonal-like” mediators of inter-kingdom communication [5]. Therefore, we focused here on the search for OMVs in the blood of healthy donors. Our study was performed on EVs isolated from the RBC concentrates of 14 healthy donors and revealed the presence of bacterial proteins and lipids similar to those present in OMVs prepared in vitro from *E. coli* (see companion paper). In addition, both the LPS- and the OmpA signals detected in EVs were correlated. We thus concluded that these signals reflected the presence of OMVs in the blood of healthy donors. Given that OmpA is a membrane protein specific to the Enterobacteriaceae family, it is likely that these OMVs were produced by the gut microflora.

To analyze the EVs of the bloodstream, we used red blood cell (RBC) concentrates, which allowed us to work with large volumes (~300 mL/bag). This blood product was separated from platelets and was also leuko-reduced during its preparation. It contained about 5% to 15% plasma for RBC preservation. The selected donors were in good health: they had volunteered to give blood and had been selected on the basis of a medical examination after answering a health questionnaire. Very importantly for our study, RBC concentrates are controlled products that do not contain contaminating bacteria.

We have not yet identified the factor(s) that impacted the presence of OMVs in the blood in 12 out of the 14 tested healthy donors. Their age ranged from 27 to 62 years old excluding a possible gut leakage described in elderly people [22]. We found no correlation between the donors’ age or gender and the presence of bacterial elements. In addition, we found no correlation between the RBC concentrates’ storage time and the presence of bacterial elements. This last result was expected as this transfusion product is supposedly bacteria free. It would be interesting to compare the antibiotic history, the food diet as well as the intestinal epithelial permeability of those blood donors to determine whether the blood EV LPS/OmpA ratio could be used as a blood marker for potential dysbiosis. Indeed, dysbiosis is linked to systemic diseases [23].

OmpA had been shown to play an important structural role in membrane stability and resistance to environmental stress in bacteria [24]. This protein may thus have the same importance for OMVs. We used this OMV marker to compare the OMV amounts between the donor RBC-EV samples (8 µg of lipids). We used bacterial OMVs as the positive and relative controls and found that 10 out of 14 EVs contained an equivalent amount of OmpA-bearing OMVs (Figure 2B). Nevertheless, one should bear in mind that the diameter of the RBC-EVs varies from that of *E coli* OMVs (40 nm, as determined in the companion paper). Therefore, the number of vesicles may vary between the two types of analyzed samples and our quantitation is thus a relative measure between RBC-EVs.

OmpA was shown to interact with the Ecgp surface receptor on brain microvascular endothelial cells in order to mediate bacterial invasion [25]. It might thus be possible that OmpA in OMVs plays a similar role in mediating the invasion of OMVs through endothelia. Both the OmpA protein and the LPS were also detected by Tulkens et al. in bacterial EVs isolated from blood samples [11]. In our study, we showed that these markers varied between healthy donors. For example, sample #6 was very rich in OMVs. The diversity of both OmpA and LPS profiles in the analyzed circulating blood samples could reflect the variable extent of Gram-negative bacteria within the gut microbiota of various individuals. Based on these results, it would be of interest to extend the investigations and seek the presence of bacterial EVs from Gram-positive-, non-Enterobacteriaceae Gram-negative bacteria as well as EVs from Fungi and Protozoa in the blood. It would be interesting to correlate bacterial and fungus EV features in the blood (LPS O-antigen, teichoic acid, peptidoglycans, membrane proteins, and 16S RNA) with eubiosis and dysbiosis. More gut microbiota vesicles are expected in the case of dysbiosis-induced leaky gut.

To bring the first evidence of passage interactions between OMVs and human cells, we focused on *E coli* OMVs. We analyzed the fusion of fluorescently labeled *E. coli* OMVs with various types of cells present among human PBMCs. We found that most of the monocytes (91.6%, Figure 3) became fluorescent. It has been shown that LPS present on the membrane of *E. coli* OMVs can be recognized by the CD14 coreceptor of TLR4 (Toll-Like Receptor 4), which is abundant in the plasma membrane of monocytes [26]. This abundance could easily explain the privileged interaction between OMVs and monocytes, this interaction being potentially sustained by multiple fusion pathways including those using a cell membrane receptor. Monocytes are specialized phagocytic cells and their ability to take up OMVs is thus not surprising. The other cell types studied here included T cells and B cells. These cells did not interact with OMVs. We also included the analysis of γδ2 unconventional T-cells because γδ TCRv2 cells are known to recognize and present bacterial components from other immune cells [27]; however, in our setting, they did not acquire any significant fluorescence from the *E. coli* labeled OMVs (Figure 3).

Three forms of LPS could be present in blood: free endotoxin, micelles, or OMVs [28]. It is known that endotoxins are proinflammatory; conversely, we suspect that OMVs as well as LPS micelles [29] are tolerated by the immune system. Therefore, it would be interesting to compare the immune role of LPS as endotoxins, micelles, or extracellular vesicles.

## 4. Materials and Methods

### 4.1. Production of E. coli OMVs

The *E. coli* clone used for this study was REL606 (see companion paper). Bacterial cells were revived overnight from glycerol stocks stored at −80 °C by starting 10 mL pre-cultures at 37 °C, 180 rotations per min (rpm), in DM1000 (Davis minimum medium: 30.6 mM K_2_HPO_4_, 15 mM KH_2_PO_4_, 6.2 mM (NH_2_)_4_SO_4_, 2 mM Na_3_C_6_H_5_O_7_, 0.83 mM MgSO_4_, 7.54 M thiamine, supplemented with 5.55 M (or 1000 mg/mL) glucose. Ten mL of the revived bacteria were transferred into a 5 L Erlenmeyer flask containing 1 L of DM1000. The flask was placed at 37 °C, under agitation at 180 rpm, until the culture reached the OD_450_ (measured using an Eppendorf (Hamburg, Germany) Biospectrometer) corresponding to the selected strain’s mid-log phase (5h 50 min, see companion paper).

Bacterial cells were pelleted by a 30 min centrifugation at 5000× *g*, 21 °C, in a Beckman Coulter (Villepinte, France) Avanti J-E JSE02C09 centrifuge. The supernatant was filtered into a clean bottle using a 0.22 µm filter (steritop Millipore, Burlington, MS, USA). The OMVs from this 1 L solution were pelleted and concentrated into eight tubes by a series of 1 h ultracentrifugation runs at 150,000× *g* (41,000 rpm), 4 °C, using a Thermofisher (Waltham, MS, USA) Sorwall WX ultracentrifuge (brake of the ultracentrifuge set to 1 to prevent the dispersion of the non-visible OMV pellets). Finally, OMVs were dispersed into 4 mL of 0.22 µm-filtrated phosphate buffered saline [PBS] solution (137 mM NaCl, 2.7 mM KCl, 10 mM Na_2_HPO_4_, 1.8 mM KH_2_PO_4_) and stored at 4 °C until further use. We measured the lipid concentration to be 5 mg/mL.

### 4.2. Preparation of Human Peripheral Blood Mononuclear Cells (PBMCs), Red Blood Cell Concentrates, and Extracellular Vesicles

The analyzed human products came from a collection of human samples that complies with the Etablissement Français du Sang (EFS)-Auvergne Rhône-Alpes (AuRA) ethics committee and is accredited by the Ministry of Education and Research under the reference AC-2020-3959. Mandatory written informed consent was obtained from all blood donors and samples were analyzed anonymously. PBMCs were purified by Ficoll-Hypaque density-gradient centrifugation (Eurobio, Ulis, France) and frozen until use.

Fourteen randomly selected red blood cell (RBC) units were kindly provided by the EFS Products for Education and Research (PLER) department. In France, the EFS blood bank follows the WHO recommendations for inclusion and exclusion criteria. For example, a donor can give blood if he or she is in good health, between the ages of 18 and 70, and weighs at least 50 kg. Excluded from donating are people with colds, flu, sore throats, cold sores, stomach problems, or any other infection, and people who have traveled to areas where mosquito-borne infections are endemic. A complete list of criteria is provided at https://www.legifrance.gouv.fr/jorf/id/JORFTEXT000042044222 (accessed on 8 November 2022). The source from which participants were recruited was random.

EVs present in transfusion units were first separated from RBCs by centrifugation. The blood bag content was transferred into 50 mL tubes, diluted with a half volume of PBS, and centrifuged for 30 min at 3200× *g*, 4 °C. The supernatant was centrifuged again for 45 min in the same conditions to remove the remaining cells and debris. Then, the supernatant was transferred to ultracentrifuge tubes, except for the 5 mL present in the conical part of the tube to avoid touching the pellet. The supernatant was centrifuged at 100,000× *g*, 4 °C, for 60 min without a break to produce a pellet containing RBC concentrate-EVs. The supernatant was carefully removed, and the EVs dispersed in PBS. Finally, the suspension containing RBC-EVs was filtered through a 0.22 μm-pore filter and stored in aliquots at −80 °C. The aliquots were thawed at 4 °C. Both the concentration and the diameter of EVs were assessed by nanoparticle tracking analysis (NTA) performed on a Nanosight NS300 (Malvern, Malvern, UK) equipped with a syringe pump. EVs were diluted in PBS to reach 20 to 60 events per frame and loaded in the syringe. The pump speed was set to 20, the camera level was at 13 and the detection threshold at 7. Five 30 s videos were captured, processed, and averaged for each EV sample using the NTA 3.0 software as described by Laulagnier et al. [30].

### 4.3. Quantification of the Lipids Present in OMVs and EVs by Measurement of the Fluorescence Emitted by Incorporated 1-Anilinonaphthalene-8-Sulfonic Acid (ANS)

The lipids present in OMVs were quantified after incorporation of ANS lipophilic molecules (Sigma, St. Louis, MO, USA) in their membrane and the measurement of their emitted fluorescence. This assay required a standard curve built with liposomes used as the reference of the assay. Liposomes were prepared from a mix of artificial lipids representing the most abundant lipids of bacterial membranes. The 16.6 mg/mL lipid stock solution was prepared by mixing 160 µL of 36 mM (25 mg/mL) phosphatidyl ethanolamine (PE; Avanti, Alabaster, AL, USA), 160 µL of 36 mM (25 mg/mL) phosphatidyl glycerol (PG), and 32 µL of 14 mM (10 mg/mL) cardiolipin (CL) in a glass tube. After drying the lipid film under a N_2_ stream, lipids were dispersed in 500 µL of a [50 mM Tris, 200 mM KCl, pH 7.5] solution, leading to the spontaneous formation of multi-lamellar vesicles. This solution was frozen in liquid N_2_, then heated four times at 50 °C, before being passed five times through an extruder (Avanti) equipped with a 0.2 µm-diameter pore filter and 19 times through the same extruder equipped with a 0.1 µm-diameter pore filter (Avanti).

An ANS assay working solution was prepared by mixing 20 µL of 0.3% ANS with 6 mL of [50 mM Tris, 200 mM KCl, pH 7.5] solution (dilution: 1/300). Two hundred µL of this ANS working solution were distributed into the wells of a black, flat-bottom 96-well plate (Corning, Corning, NY, USA), to which 1, 2, or 3 µL of the 1 mg/mL liposome solution, or 1 µL of the OMV or EV solution were added. Each vesicle sample (liposomes, OMVs or EVs) was triplicated. The fluorescence was measured using a Varioskan fluorimeter (Thermofisher) after setting up the excitation wavelength at 350 nm and the emission wavelength at 512 nm (100 ms time reading frame).

### 4.4. Dot Blots

Following the purification of EVs, the proteins and associated lipopolysaccharides (LPS) were analyzed by dot blot on a nitrocellulose membrane (Bio-Rad, Hercules, CA, USA). Eight µg (lipid amount) of each type of EVs were spotted. The membrane was blocked for 30 min in 5% non-fat dry milk in [50 mM Tris, 200 mM NaCl, pH 7.5, 0.1% Tween-20] (TBS-T), then incubated overnight in 2.5% non-fat dry milk in TBS-T containing either mouse monoclonal anti-*E. coli* LPS IgGs (clone 2D7/1, ab35654, Abcam, Cambridge, UK) diluted 1/2000 or rabbit anti-OmpA polyclonal antibodies (Epigentek, East Farmingdale, NY, USA) diluted 1/2000. After two washes of 10 min each in TBS-T, the membranes were incubated for 3 h, respectively in 2.5% non-fat dry milk in TBS-T containing anti-mouse polyclonal antibodies coupled to horse radish peroxidase [HRP] and diluted 1/10,000 or containing HRP-conjugated anti-rabbit IgG F(ab′)_2_ fragment (Sigma) diluted 1/10,000. After washes in TBS-T, the membranes were revealed by chemiluminescence using a Clarity Western substrate kit (Bio-Rad) and photographed using a ChemiDoc apparatus (Bio-Rad).

### 4.5. Immunoblots

EV solutions containing 8 µg of lipids were mixed with 4xLaemmli sample buffer (Bio-Rad). The mixtures were denatured for 10 min at 95 °C, loaded into the wells of a 12% polyacrylamide gel calibrated with 5 µL of Precision Plus Protein™ All Blue Prestained Protein Standard molecular weight marker (Bio-Rad). The proteins were separated for 1 h 30 min at 30 mA, 100 V. Following SDS-PAGE, the proteins and associated lipopolysaccharides (LPS) were transferred from the polyacrylamide gel to a nitrocellulose membrane (Bio-Rad) using a Trans-Blot turbo apparatus (Bio-Rad) according to the manufacturer’s recommendations. The membrane was blocked for 30 min in 5% non-fat dry milk in TBS-T, then incubated overnight in 2.5% non-fat dry milk in TBS-T containing either rabbit anti-Actin (ab8227S, AbCam, Cambridge, UK), rabbit anti-Alix (#18269S, Cell Signaling, Danvers, MA, USA), or rabbit anti-CD63 (ab216130, AbCam), all diluted 1/1000 and mouse monoclonal anti-*E. coli* LPS IgGs (clone 2D7/1, ab35654, Abcam) diluted 1/2000. After two washes of 10 min each in TBS-T, the membranes were incubated for 3 h in 2.5% non-fat dry milk in TBS-T containing either anti-rabbit IgG F(ab’)_2_ fragment (Jackson Immunoresearch, West Grove, PA, USA) diluted 1/10,000 or anti-mouse polyclonal antibodies coupled to HRP and diluted 1/10,000. After washes in TBS-T, the membranes were revealed by ECL using a Clarity Western substrate kit (Bio-Rad) and photographed using a ChemiDoc apparatus (Bio-Rad).

### 4.6. Fusion Experiments between OMVs and Peripheral Blood Mononuclear Cells

OMVs (corresponding to 200 µg of lipids) were incubated in 1 mL of PBS supplemented with 5 µL of the far-red fluorescent, lipophilic carbocyanine DiD (DiIC18(5) solid (1,1′-Dioctadecyl-3,3,3′,3′-Tetramethylindodicarbocyanine, 4-Chlorobenzenesulfonate Salt, Invitrogen, Waltham, MA, USA) for 1 h at 20 °C. After 3 h of centrifugation at 20,000× *g*, 4 °C, the labeled pellet was dispersed in 50 µL of PBS and filtered (0.22 µm). Five µL of the labeled OMVs (5 10^10^ OMVs) were incubated with 2 × 10^5^ PBMCs for 16 h at 37 °C, 5% CO_2_, in incubation tubes. After centrifugation of the cells at 800× *g* for 5 min (4 °C), free OMVs were removed and cell pellets were washed with PBS. PBMC pellets were dispersed in 1 mL PBS and the cell fluorescence was analyzed using a FACS Canto II (BD Biosciences, Haryana, India) flow cytometer. The analysis was performed on singlet cells defined by FSC-A/FSC-H (forward scatter -amplitude and -height) and SSC-A/SSC-W (side scatter -amplitude and -height). The cells were then gated on FSC-A/SSC-A and typed using fluorescently-labeled anti-CD3 (T-cells), anti-CD14 (monocytes), anti-CD19 (B-cells), anti-TCR gamma/delta (Tγδ), and anti-TCR-V delta2 (Tγδ vd2 subtype) antibodies from BD Biosciences.

## 5. Conclusions

This work provides evidence that microbiota-derived OMVs are commonly present within the hosts’ circulating blood. This discovery paves the way for novel studies that will dissect the interactions between OMVs and eukaryotic cells as well as the transcytosis of OMVs throughout cellular barriers. We assume that bacterial EVs participate in both physiological and pathological processes, as an integral part of microbiota communication signals. Therefore, studying the journey of bacterial EVs through the body, especially their relations with the immune system, would contribute to a new understanding of the essential functions of the gut microbiota. This EV-based relation is a pioneer concept [10,13] providing new research directions to study eubiosis and dysbiosis. In order to gain further insights, we are now studying bacterial EV/immune system interactions in the context of ex vivo experiments on human blood as well as their putative impact in human transfusions. These studies should expand our understanding of the interplay between the microbiota and the human immune system and provide fundamental data necessary for the development of OMVs as biotechnical tools for the targeted delivery of drugs against cancer [5] and neurological disorders [4]. Finally, exploiting OMVs’ properties for therapeutic purposes appears to be very attractive, notably because they are not capable of self-replication, contrary to live bacteria, thus reducing the biosafety risks. One could thus propose the use of oral OMVs, derived from beneficial bacteria, in treating various kinds of metabolic and immunological disorders.

## Figures and Tables

**Table 1:** Characteristics of the EVs from RBC concentrates of healthy donors. The age, gender, and blood group of the blood donors are indicated as the storage time of the RBC concentrates. The EV concentration, their mean diameter, and the mode (major peak) were obtained by NTA.

Blood Donor EV	Concentration (EV/mL)	Mean Size (nm)	*±SD*	Mode (nm)	Storage Time (days)	Blood Group	Gender	Age
#1	1.09 × 10^10^	126.4	*12.9*	87.4	42	AB^+^	M	48
#2	3.90 × 10^11^	183.3	*0.6*	183.5	9	A^+^	F	27
#3	5.93 × 10^11^	192.3	*1.1*	198.9	12	O^−^	F	39
#4	4.25 × 10^11^	180.7	*0.1*	168.2	15	O^−^	M	63
#5	2.06 × 10^11^	180.4	*1.5*	157.9	15	O^+^	F	33
#6	3.85 × 10^12^	178.7	*1.3*	154.9	38	O^+^	M	62
#7	1.05 × 10^15^	183.6	*1.3*	174.1	21	A^+^	M	35
#8	9.91 × 10^11^	167.7	*1.3*	154.3	31	O^+^	M	63
#9	9.75 × 10^10^	171.8	*2.5*	161.0	10	A^+^	M	51
#10	4.27 × 10^11^	157.4	*0.8*	140.2	20	AB^+^	F	36
#11	7.26 × 10^11^	181.4	*1.4*	186.1	27	A^−^	M	55
#12	4.99 × 10^11^	166.3	*0.4*	141.8	30	O^+^	M	35
#13	3.94 × 10^11^	161.8	*1.0*	144.0	31	O^+^	M	55
#14	6.03 × 10^11^	161.7	*0.9*	133.4	36	O^+^	F	30
Mean	7.56 × 10^13^	171.0		156.1	24.1			45
SD	2.80 × 10^14^	16.4		27.5	11.0			13
Min.	1.09 × 10^10^	126.4		87.4	9.0			27
Max.	1.05 × 10^15^	192.3		198.9	42.0			63
